# Different Sources of Threat on Math Performance for Girls and Boys: The Role of Stereotypic and Idiosyncratic Knowledge

**DOI:** 10.3389/fpsyg.2016.00637

**Published:** 2016-04-29

**Authors:** Isabelle Régner, Leila Selimbegović, Pascal Pansu, Jean-Marc Monteil, Pascal Huguet

**Affiliations:** ^1^Laboratoire de Psychologie Cognitive, UMR 7290, Centre National de la Recherche Scientifique, Aix Marseille UniversitéMarseille, France; ^2^Center for Research on Cognition and Learning, Centre National de la Recherche Scientifique UMR 7295, University of PoitiersPoitiers, France; ^3^Laboratoire des Sciences de l'Education, Université Grenoble AlpesGrenoble, France; ^4^Laboratoire de Psychologie Sociale et Cognitive, Centre National de la Recherche Scientifique UMR 6024, Université Blaise PascalClermont-Ferrand, France

**Keywords:** stereotype threat, idiosyncratic effect, personal academic experiences, math performance, children

For 20 years, the impact of stereotypical knowledge on math performance has been intensively investigated, especially within the framework of “stereotype threat” (Steele, 1997). Stereotype threat (ST) theory and research “do not focus on the internalization of inferiority images or their consequences. Instead, they focus on the immediate situational threat that derives from the broad dissemination of negative stereotypes about one's group—the threat of possibly being judged and treated stereotypically, or of possibly self-fulfilling such a stereotype” (Steele and Aronson, 1995, p. 798). Here, we distinguish between ST and another powerful yet relatively neglected factor in the determination of math performance: self-images of inferiority derived from personal history of failure. There is some evidence that such self-images of inferiority may also lead to under performance in math tests (hereafter referred to as idiosyncratic effects). One question that arises is whether and how ST and idiosyncratic effects interact with each other, which would offer a fuller picture combining the intervention of stereotypic and idiosyncratic knowledge in math performance.

## ST effects

ST refers to a decrease in test performance in situations where individuals feel threatened by the possibility that their performance will confirm—to others, and/or themselves—a negative stereotype about their group abilities (Steele, 1997). This situational threat increases concern about being stereotypically judged and mistreated, which impairs processing efficiency and leads to underperformance (Schmader and Johns, 2003). Consistent with this, females underperform relative to equally qualified males on difficult math tests when told that the test is gender-biased or when simply told that it measures math skills, but perform as well as males when told that the test is gender-fair or when it is supposedly not diagnostic of math abilities (Spencer et al., 1999; for reviews see Ben-Zeev et al., 2005; Régner et al., 2014).

ST typically affects only a sub portion of the stereotyped group, those with the skills and self-confidence to have identified with the domain (Steele, 1997). On the contrary, less confident and less identified individuals, those who have internal doubts about their ability, are likely to underperform regardless of whether they are stereotype threatened in the situation. Consistent with this, ST effects have been mostly examined and found among high achieving females majoring in Math, Science, and Engineering (Spencer et al., 1999; Bell et al., 2003; Good et al., 2008; Régner et al., 2010) and high math-identified females (Cadinu et al., 2003; Keller, 2007). The myriad studies conducted since Steele and Aronson's (1995) seminal paper clearly demonstrate the influence of stereotypical knowledge in the math domain.

## Idiosyncratic effects

If the influence of students' inferiority images derived from their own failures in math was not in the scope of stereotype threat theory, support for this idiosyncratic influence can be found in the literature on autobiographical memory. Some studies indicate that memories of personal academic successes or failures can be activated by the testing situation and then impact one's current performance (Monteil and Huguet, 1993, 1999). Monteil (1988, 1991) showed that students with past failures in math (low achievers) who publicly received a positive feedback on a preliminary math test obtained lower performance on a subsequent test when it was taken in a public rather than private context, as if they could not publicly deal with a positive feedback. The reverse pattern was obtained for high achievers having received positive feedback who then underperformed in the private (rather than public) context. In general, students facing inconsistencies between their own academic history and the testing situation (e.g., low achievers receiving positive feedback) are more self-focused, resulting in impaired task performance (Monteil et al., 1996; Brunot et al., 2000). In Huguet et al. (2001), students with past failures or successes (low vs. high achievers) in math were asked to learn a complex figure, and to reconstruct it from memory on paper. They were either told the test would measure their ability in geometry or in drawing. Whereas, low achievers underperformed relative to high achievers in the geometry condition, low and high achievers performed equally well in the drawing condition. Low achievers' performance was thus inhibited when the task characterization referred to a domain associated with past generalized failures while the test was exactly the same in both contexts.

Selimbegović et al. (2011) went a step further by activating and measuring autobiographical memories of success vs. failure, while distinguishing between general and specific memories. Before taking a math test, participants had to recall three general vs. specific autobiographical memories of either their past academic successes or failures. General memories of failure and specific memories of success resulted in worse math performance than general memories of success and specific memories of failure. Additionally, general memories of failure and specific memories of success induced fear of failure (Selimbegović et al., 2011) or threat appraisal (Selimbegović et al., 2015), with increased fear of failure playing a mediating role in performance. In sum, knowledge about one's past academic performances can induce counterproductive self-focus, fear of failure, threat appraisal, and impaired performance. This is enough evidence to consider idiosyncratic knowledge as another potential threat for math performance.

## Current research

Whether and how ST and idiosyncratic effects interact remains unexplored. We examine this issue with a reanalysis of Huguet and Régner's (2009) ST study. Compared with the other studies reported above, this research has the advantage to provide all necessary measures to simultaneously test ST and idiosyncratic effects. It comprised both male and female participants (the sex-ratio of samples used in previous “idiosyncratic effects” studies did not allow to test for ST), used the same geometry/drawing paradigm as in Huguet et al.'s (2001), comprised students' math grades and a measure of their perceived personal reputation in terms of “good” vs. “bad” students in math.

Consistent with ST theory, Huguet and Régner (2009) found a significant gender by task characterization interaction: whereas girls underperformed compared to boys in the geometry condition, they outperformed boys in the drawing condition (See also Huguet and Régner, 2007). Assuming that ST and idiosyncratic effects interact with each other, low-achieving girls in the geometry condition (cumulating the threats related to their own personal academic experiences and gender group) would obtain the worst performance. However, this hypothesis is hardly compatible with ST theory that predicts ST to have its greatest effect on the better, more confident students in stereotyped groups. An alternative could be that ST and idiosyncratic effects do not interact but occur simultaneously. This would imply the coexistence of both effects in the same data set: girls underperforming relative to boys in the geometry condition, while outperforming them in the drawing condition, and the low achievers (both genders) underperforming relative to high achievers in the geometry condition, while performing equally well as them in the drawing condition.

## Further analysis of Huguet and Régner's (2009) data

The participants were 199 French middle-school students (92 girls and 107 boys, mean age = 12.12, *SD* = 0.70). Like in Huguet et al. (2001), they had to learn a complex figure (made of 22 units) and then to reconstruct it from memory on paper. Students were either told the test would measure their ability in geometry or in drawing. Recall performance was measured in terms of both the number and quality of the units reproduced from the complex figure. Two points were given if the unit was correct and properly positioned, 1 point if it was either altered but correctly placed or not altered but incorrectly placed, 0.5 point if it was altered and in a wrong place, and 0 if it was missing or unrecognizable. The possible scores could range from 0 to 44 (Grand Mean = 23.37; *SD* = 6.09; *min* = 4.50 and *max* = 40). Students' math grades were available from the school records on a scale ranging from 0 to 20 (Grand Mean = 12.15; *SD* = 3.89; *min* = 2.30 and *max* = 19). The present reanalysis required using Task characterization, students' Gender and Math grades as predictors in order to test ST effects (Gender × Task characterization interaction), idiosyncratic effects (Students' grades × Task characterization interaction), and the three-way interaction.

Since, contrary to Huguet et al. (2001), participants in Huguet and Régner (2009) had not been selected a priori on the basis of their achievement level in Math, it was important here to make sure that those with lower vs. higher math grades were aware of their inferiority vs. superiority in this domain. For that purpose, we used Huguet and Régner's (2009) measure of students' perception of their personal reputation in terms of “good” vs. “bad” student in Math within their class. Students answered two items: “Among your classmates, how many think you're a good student in Math ?” (item 1) and “How many classmates think you are not good in Math ?” (item 2), using a 5-point Likert scale ranging from 1(none) to 5 (everybody). These items were subtracted to distinguish between students considering they had a good or bad reputation in math and those considering they were average or with no specific reputation. Any score different from zero means that students considered they had either a relatively good or bad reputation, which was the case of most participants (63.3%). Participants whose perception of personal reputation was average or unclear (score equal to zero), were removed from our reanalysis. Therefore, the distinction between low vs. high achievers did not rely exclusively on students' math grades but also on students' perception of their personal reputation in math, while excluding both average students and those reporting no clear personal reputation in that domain. The final sample included 126 participants, with 57 girls (25 in the Geometry condition and 32 in the Drawing condition) and 69 boys (41 in the Geometry condition and 28 in the Drawing condition).

## Results

**Figure 1:**
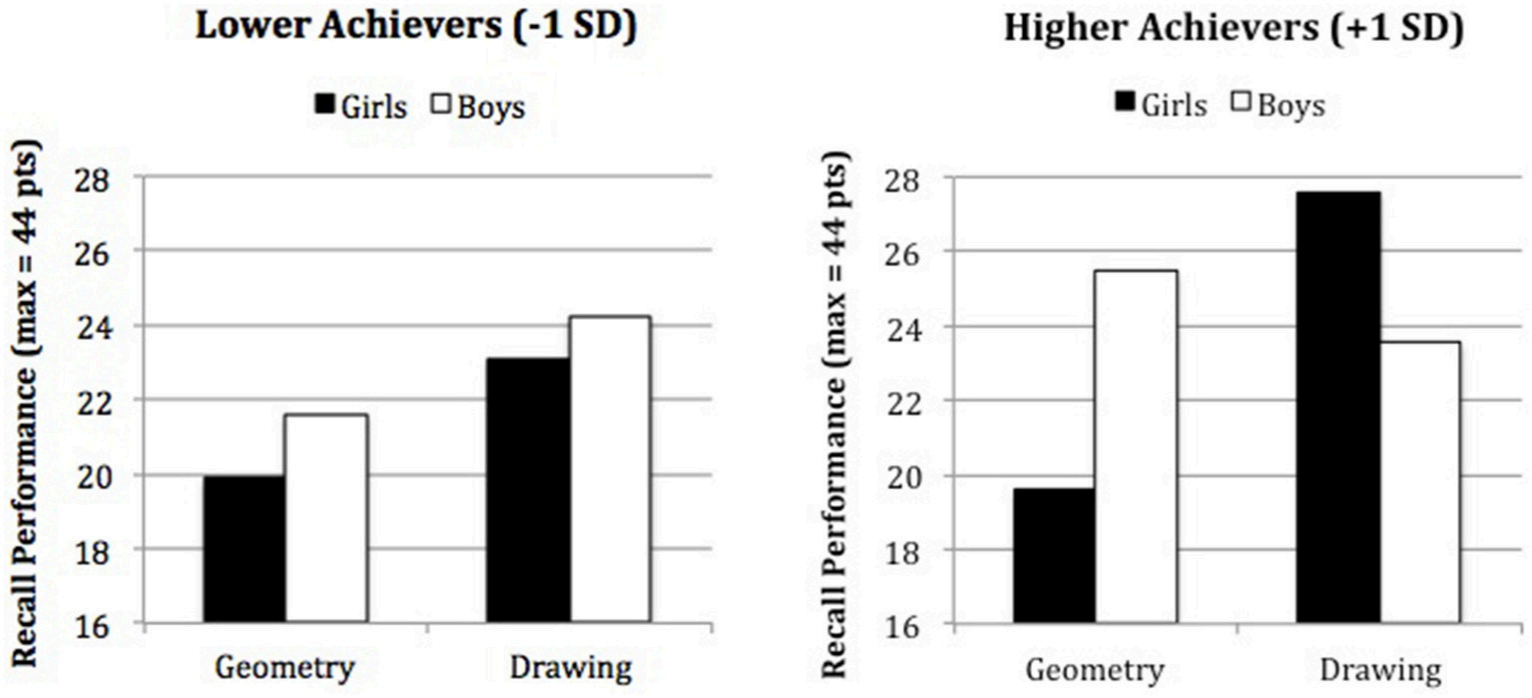
**Recall performance as a function of Gender and Task Characterization for lower (−1 *SD*) and higher (+1 *SD*) achievers in Math**.

We regressed students' recall performance on gender (boys = 0, girls = 1), task characterization (drawing = 0, geometry = 1), math grades (mean-centered), and their interaction terms. The Gender by Task characterization interaction was significant, indicating the presence of a ST effect unfavorable to girls [(Huguet and Régner, 2009), *b* = −5.22, *SE* = 2.08, *t*_(117)_ = −2.50, *p* = 0.014]. Although, the Students' grades by Task characterization interaction did not reach significance [*b* = 0.58, *SE* = 0.38, *t*_(117)_ = 1.53, *p* = 0.128], the three-way interaction did, *b* = −1.19, *SE* = 0.55, *t*_(117)_ = −2.17, *p* = 0.032 (Figure 1). We used Preacher et al.'s (2006) interactive calculation tools to probe this interaction by estimating simple slopes at low (−1 *SD*) and high (+1 *SD*) values of our continuous predictor (math grades). We also used Keppel's modified Bonferroni correction to control for error rate with planned comparisons (Keppel, 1991), which led to consider only two simple slopes as significant. In line with ST theory, the higher math grades, the stronger ST effect: girls underperformed relative to boys in the Geometry condition, *b* = −5.83, *SE* = 2.12, *t*_(117)_ = −2.75, *p* = 0.007, whereas girls and boys performed equally well in the drawing condition. An idiosyncratic effect also occurred but only among boys: the lower achievers underperformed relative to the higher achievers in the geometry condition, *b* = 0.49, *SE* = 0.22, *t*_(117)_ = 2.29, *p* = 0.024, whereas the lower and higher achievers performed equally well in the drawing condition.

## Discussion

The present findings provide first evidence that both stereotype threat and idiosyncratic effects can occur in children without cumulative effects: stereotype threat occurred among high-achieving-girls, while the idiosyncratic effect occurred in low-achieving boys. Using math grades as a moderator (rather than a covariate to adjust the outcome for prior performances or an inclusion criterion to select talented students as it is usually the case in stereotype threat studies), we found that stereotype threat is more likely in girls with higher math grades. This result is consistent with Steele's (1997) basic argument that stereotype threat typically affects the higher achievers, those with the skills and self-confidence to have identified with the domain. Interestingly, neither students' achievement level nor their domain identification were taken into account in recent meta-analyses (Stoet and Geary, 2012; Flore and Wicherts, 2014) that downplayed the seriousness of ST effects. Although, we agree with these papers that the importance of ST effects should not be overstated, we also think that the key moderators of these effects should not be underestimated either. In their meta-analytic review, Walton and Cohen (2003) clearly found that both ST (as well as stereotype lift effects) are much more likely among stigmatized who are high achievers and/or highly identified with the domain. Consistent with this, in our own female sample the higher the math grades, the higher ST effect.

On the contrary, why girls did not experience idiosyncratic effects is difficult to explain. The negative math-gender stereotype is so powerful that it may have overcome the influence of other sources of threat like inferiority images derived from one's personal academic experiences. Girls' self-construal being mostly interdependent and boys' self-construal mostly independent (Markus and Kitayama, 1991; Huguet and Monteil, 1995; Keller and Molix, 2008), girls may be especially sensitive to collective reputations and boys to personal reputations. Although, the dissociation found here between stereotype threat and idiosyncratic effects needs to be better understood, it seems that inferiority images rooted in stereotypic vs. idiosyncratic knowledge are different sources of threat on math performance. This is an important conclusion as the exact relationships between ST and other sources of threat such as self (rather that group)-images of inferiority in math (or other domains) remained unexplored so far. In line with ST theory (Steele, 1997), ST research indeed focused on high achievers and neglected those with self-images of inferiority (i.e., low achievers). However, in parallel, some studies in the past 25 years provided evidence that self-images of inferiority also lead to underperformance in math tests (e.g., Monteil and Huguet, 1999; Selimbegović et al., 2011, 2015). The time has come to integrate both literatures. The present re-analysis is a first step in this direction.

## Author contributions

All authors listed, have made substantial, direct and intellectual contribution to the work, and approved it for publication.

### Conflict of interest statement

The authors declare that the research was conducted in the absence of any commercial or financial relationships that could be construed as a potential conflict of interest. The reviewer LC and handling Editor declared their shared affiliation, and the handling Editor states that the process nevertheless met the standards of a fair and objective review.
